# Hip Arthropathy in Haemophilia

**DOI:** 10.3390/jcm6040044

**Published:** 2017-04-08

**Authors:** Christian Carulli, Anna Rosa Rizzo, Massimo Innocenti

**Affiliations:** Orthopaedic Clinic, University of Florence, Florence 50139, Italy; annar.rizzo@gmail.com (A.R.R.); m.innocenti@med.unifi.it (M.I.)

**Keywords:** physical therapy, viscosupplementation, synoviorthesis, total hip artrhoplasty, haemophilia, hip, ceramics, ceramic on polyethylene coupling, cementless arthroplasty

## Abstract

Hip arthropathy in haemophilic patients is disabling for hip and other common target joints. Even if bleedings in the hip are not frequent, femoroacetabular alterations may affect the functional ability of patients at a very young age. A haematologic prophylaxis combined with an adequate lifestyle and regular and low-traumatic physical activity are the keys to preventing such arthropathy. In the early stages of arthropathy, anti-inflammatory drugs and physical therapy may be sufficient to limit its progression. In cases of recurrent symptoms, viscosupplementation with hyaluronic acid, and chemical synoviorthesis are useful options. In more advanced stages, hip arthroscopy may be treated by synovectomy or loose body removal. For late stages, total hip arthroplasty (THA) is mandatory. Until a few decades ago, the clinical outcomes after hip arthroplasty were variable, due to the different management of patients and the use of old generation implants and couplings. In the last decade, the introduction of the multidisciplinary management and the use of modern cementless implants with high performing materials and less invasive surgical techniques have dramatically improved the functional results. Nowadays, as is the case for other target joints, the purpose of the management in haemophilia centers is the early detection of any hip alterations—by clinical and ultrasound (US) evaluations of patients in childhood—to reveal any early articular damage and to provide adequate treatment in case of symptoms. The present paper represents an updated review of the several approaches to hip arthropathy in haemophilia.

## 1. Introduction

The hip is uncommonly affected by recurrent bleeding related to haemophilia. The main reasons are related to the paucity of synovial tissue and the lower risk of trauma with respect to other target joints. However, hip arthropathy in persons with haemophilia is poorly tolerated—similar to other target joints [1]. Primary prophylaxis in subjects with moderate to severe factor deficiency should be sufficient to prevent bleedings in the hip. However, persons with mild or moderate haemophilia may develop arthropathy.

Hips are “ball and socket” joints characterized by wide mobility and a high tendency to wear in the case of disease. A series of bleedings may affect the integrity of the chondral layer surrounding the femoral head or the acetabular labrum attached to the cartilage, leading to the global deterioration of the joint. Recurrence of haemarthrosis induces the proliferation of a small amount of synovial tissue, and hypertrophy of such tissue creates a vicious cycle ending with the so-called “haemophilic arthropathy” [2].

As for other articular conditions, several stages of arthropathy may be found and it is crucial to prevent the disease or at least detect the early stages of it to stop or limit its progression.

Ankles, knees, and elbows represent the first joints affected in haemophilic children during the development of their initial movements and gait ability at about one year of age. Hips are generally affected at a later stage, mostly when children begin to run or jump.

**Figure 1:**
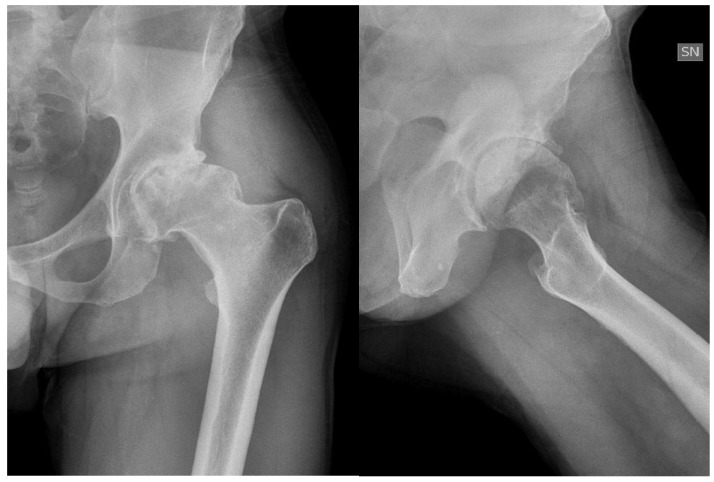
Forty-six-year-old haemophilic subject affected by severe haemophilia A, with inhibitors, with symptomatic left hip arthropathy secondary to femoral acetabular impingement.

The modern management of haemophilia is usually deemed to be sufficient to prevent any form of hip arthropathy, except in the case of inhibitors. However, patients with recurrent bleeding, with a history of trauma or with associated femoroacetabular impingement are prone to develop haemophilic arthropathy (Figure 1).

The early stages of arthropathy may be detected by ultrasound (US) examination in children and very young subjects, at least during the first orthopaedic evaluation, even though the hip does not represent a typical target joint. In patients showing hip alterations, it is useful to perform periodic US examination to detect blood in the joint, synovitis, and early chondral gaps for elbows, knees, and ankles. In these conditions, the prevention of traumatic injury, the limitation of highly traumatic activities, and the prescription of non-contact sport or physical activity are useful.

In young adults, US may not be useful, while standard X-rays may be performed to assess the stage of arthropathy, based on the well-known criteria of Pettersson [3] or Gilbert [4].

Once a diagnosis has been made, and depending on the symptoms expressed by the patients, a treatment should promptly be prescribed. 

The following is a brief overview of the modern management of hip arthropathy in haemophilic patients.

## 2. Conservative Treatment

Historically, total hip arthroplasty (THA) has been considered the only successful treatment of hip arthropathy in haemophilia. However, several conservative approaches may be considered as useful in the hip, as well as for other joints [5,6,7]. By an early US detection of joint alterations, often asymptomatic during childhood, we can nowadays use non-invasive methods for the treatment or prevention of symptomatic hip arthropathy.

**Figure 2:**
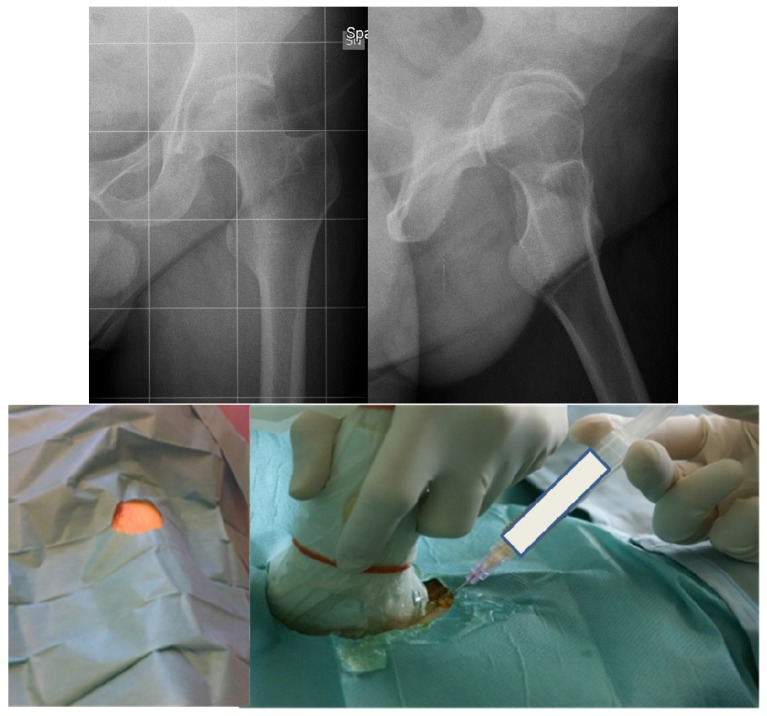
Radiograms of a forty-eight-year-old haemophilic subject affected by severe haemophilia A with symptomatic left hip arthropathy treated by hyaluronic acid intraarticular injections in a sterile procedure.

The haematological protocol with supplementation of the deficient factor is mandatory, if possible by a primary prophylaxis. In the case of arthropathy, in the early stages, a secondary prophylaxis is crucial to prevent the progression of arthropathy. As previously mentioned, the limitation of highly traumatic activities and the prescription of low-impact sports (mainly cycling and swimming) are proposed to improve the health status and global performance without loading the joints involved by haemophilia. Also, muscular toning exercises and stretching activity may be useful in young subjects to improve their functional ability or to recover it after a bleeding event. Anti-inflammatory drugs may be helpful in teenagers or adults combined with physical therapy. In the case of persistent symptoms (typically in the presence of active synovitis) and mild or moderate stages of arthropathy, something should be done to break the vicious circle leading to arthropathy. Intraarticular injections seem to provide good clinical effects and improve joint function in the hip, as well as in other target joints [5,6,7]. Several types of injections may be proposed: viscosupplementation by hyaluronic acid (HA); chemical synoviothesis by rifampicin or other antibiotics; radiosynoviorthesis by radionuclides; and injection with corticosteroids. Each drug has a specific purpose. HA is a polysaccharide containing glucosamine and glucuronic acid which are normally produced by synovial cells. Its effects are joint lubrication, shock absorption, and viscoelasticity of synovial fluid, but also stimulation of endogenous hyaluronic acid, anti-inflammatory effect, and inhibition of the degradative action of matrix metalloproteinases [8]. Viscosupplementation is usually proposed in symptomatic early stages of arthropathy when US examination excludes the presence of active synovitis in patients of all ages. Several experiences have been reported decades ago with encouraging short-term outcomes [9,10,11]. However, only one group proceeded with this approach in the long term, obtaining positive outcomes [5,6]. Viscosupplementation may also be considered for the treatment of hip disease with one to three injections (one per month), using US as a guide from the anterior aspect of the thigh in order to prevent any vascular or neurologic lesions (Figure 2).

In the case of active chronic synovitis, patients’ symptoms are more pronounced and tendency to bleed is generally higher than in subjects with simple arthropathy. In such conditions, after the failure of the medical and physical therapies, synoviorthesis may be useful given the hypertrophic synovium’s action of inducing fibrosis. Synoviorthesis can be performed by injections of antibiotics or radiocolloids, and it showed acceptable outcomes ranging from 76% to 80% [11]. A particular, but important future perspective, is represented by the use of synoviorthesis for patients with inhibitors.

Chemical synoviorthesis is obtained by the use of cheap antibiotics, such as rifampicin or oxytetracicline chloridrate. Caviglia and his group reported that the use of rifampicin is more effective in small joints (elbows and ankles) than large joints (knees) [12]. Fernandez-Palazzi and colleagues reported satisfactory results in terms of pain relief and functional recovery in all target joints [13]. Athanassiou-Metaxa and colleagues, in their preliminary experience with rifampicin, recorded variable results, complications (local tenderness, swelling), and a low compliance to treatment in children, particularly after rifampicin injections [10]. Similar outcomes and complications were reported by Molho and colleagues [14].

Radiosynoviorthesis is the intraarticular injections of beta-emitters drugs, which by interaction with local phagocytic cells may induce synovial fibrosis by a reduction of its vascular supply. First introduced for the treatment of several rheumatic diseases [15], it was then used for haemophilia by Ahlberg and colleagues [16]. The rationale was that radiations would cause a reduction of the synovial vasculature, consequently preventing the progression of arthropathy. The more commonly used radiocolloids are Yttrium 90 and Rhenium 186, in large and small joints respectively [17]. Recently, brand new radioactive drugs have been introduced with more rapid action and low invasiveness expected: Erbium 169 and Samarium 153. Detractors of radiosynoviorthesis address the potential stochastic effect of radiations as a contraindication of the use of such a procedure. On the other hand, authors that have used such a technique for a long time did not report any toxic or teratogenic effect on treated patients [18,19,20].

Even if never reported, there is emerging interest in tissue engineering techniques, such as the use of platelet-rich-plasma (PRP): It consists of the centrifugation of an amount of patients’ blood and extraction of the active fraction. Given the richness of growth factors in platelets, the rationale is to inject a quote of such concentration in a joint a quote of such factors to induce a “regeneration” of intraarticular tissues. However, despite the enthusiasm in recent years, there is currently no evidence to support the use of PRP in the treatment of musculoskeletal diseases and haemophilic arthropathy in the hip and other target joints [21].

## 3. Surgical Treatment

The hip is an extremely congruent joint: this implies that in the case of moderate to severe arthropathy, symptoms are likely to worsen more quickly with respect to other target joints that are poorly congruent. Moreover, in contrast to poorly congruent joints, conservative strategies are less useful and have only short-term effects. Thus, surgery for moderate to severe hip arthropathy is recommended to achieve long-term good clinical results.

Hip arthroscopy is a well known but still not globally diffuse technique: in haemophilia, it has seldom been used. It has been used for synovectomy in selected cases of moderate stages of arthropathy [22,23]: however, is should not be recommended given the risk of haematoma and bleeding related to the leg traction, which is mandatory to perform the procedure.

Historically, severe stages of arthropathy have been treated by hip replacement, associated with acceptable rates of success, despite the high risk of complications mostly related to coinfections (hepatitis C―HCV, human immunodeficiency virus―HIV), liver disease, and septic or aseptic loosening [24,25,26,27]. These variable outcomes have been related to the type of implants and couplings available at those times, and to the management of persons with haemophilia before the introduction of modern substitutive therapy. Moreover, the lack of a multidisciplinary approach increased the risk of failure of the small series reported in the literature.

During the last years, several larger series of persons with haemophilia undergoing THA with modern implants and managed by dedicated teams have been reported. Excellent outcomes and a lower rate of complications have been the goals of the multidisciplinary approach to severe hip arthropathy in haemophilic subjects [26,27,28,29,30,31]. Such studies have several common features that represent a “previous” generation of hip surgery: most authors used cemented monoblock implants, metal-on-polyethylene (MOP) couplings, small diameter heads, and quite long stems. In the last two decades, modular cementless implants were used, characterized by bioactive surfaces for a more efficient osseointegration, with ceramic-on-ceramic (COC) or ceramic-on-polyethylene (COP) bearings, and are associated with the use of shorter stems and larger femoral heads [1,31,32,33,34]. Metal-on-metal (MOM) couplings were used for a number of years but stopped being considered given the several reports on their potential biologic local and systemic sequelae [33]. With these improvements, better results and longer survival rates are expected, allowing these young subjects to maintain their primary implant for a longer period and, in the case of revision, making the removal of the components easier [1].

Carulli and colleagues were the first to report a series of 23 haemophilic patients affected by severe hip arthropathy and treated by cementless COP THA. Followed up by a multidisciplinary team with a mean follow-up of 8.1 years, they did not record failures or complications and reported satisfaction and improvements in all patients [1].

Panatopoulos et al. later reported their experience of MOM and COP THA in patients with haemophilia and other bleeding disorders with a mean follow-up of 10.4 years: while the MOM group showed a very low implant survival rate (22.2% after 18 years), the COP group presented a 100% survival rate after 7 years [32]. 

Lee and colleagues reported a series of 21 cementless THA in 17 haemophilic patients followed up at least ten years later: three revisions and one case of pseudotumor were recorded [33].

Strauss et al. recently reported their experience of 43 haemophiliacs undergoing cementless THA with a mean follow-up of 11.5 years: they recorded three infections (6.1%) and five mechanical loosenings (10.2%) needing revision; and three further surgical procedures for two cases of pseudotumor and one of periarticular ossifications [34].

## 4. Total Hip Arthroplasty: Technical Features

In a multidisciplinary perioperative theatre, after obtaining the patient’s information and acquisition of his/her consent, THA is performed by procedures that are common worldwide: general anaesthesia, antibiotic prophylaxis, tailored substitutive prophylaxis, and close survey for the first two weeks.

As for other diseases, THA in haemophiliacs may be performed by two main surgical approaches: the lateral/anterolateral approach and the posterolateral approach. They both have advantages and drawbacks, but the most important factor is the surgeon’s experience. It is well known that the lateral approach is easier and associated with a lower risk of postoperative instability with respect to the posterolateral approach. On the other hand, the posterolateral approach is more conservative and no muscular impairment is left after surgery compared to the lateral approach: in the lateral approach, a limp may be present due to abductor muscles deficiency [35,36].

As mentioned before, one of the key modern elements is surely the choice of implant. For decades, THA in haemophilia was cemented, with small-diameter metallic heads coupled with polyethylene. Later, only few experiences with MOM cementless THA were reported due to high rates of failure [32]. Most of persons with haemophilia were treated by COP and COC hip arthroplasties [1,33,34]. Given the young age of persons with haemophilia, it is reasonable to use cementless high-performance couplings because younger patients have higher functional demands, and a low wear is desirable. Furthermore, in the case of revision (rather probable for young subjects) a cementless THA is easier to remove with respect to a cemented implant. 

Short stems have recently been introduced in the treatment of haemophilic arthropathy of the hip with comparable and satisfactory results with respect to standard stems [1].

Intraoperative technical features have to be strongly considered in haemophilic patients. In such subjects, it is not easy to ensure an adequate limb length given the high probability of other target joints being involved. Flexion contractures of both knees, secondary scoliosis due to postural changes, the stiffness of ankles, and compensatory pelvis tilting are frequently present, making it hard to realize an adequate limb length and an ideal hip centre restoration. However, a mild limb length discrepancy is rarely a source of complaint by persons with haemophilia [1].

Drains are generally used and removed between 12 h and 24 h after surgery. Laboratory samplings of the deficient factor are usually performed every day for the first days, and after a week in the case of actual necessity to diminish the doses of the haematologic drugs progressively. Tailored rehabilitative protocols and dedicated physical therapy are mandatory to achieve an early recovery and muscular tone after THA. An in-patient intensive rehabilitative period should be strongly considered to allow a multidisciplinary close evaluation, as is the case for the most common knee surgery [37]. The mean characteristics of such a program are tailored pain management in the first day after surgery; exercise sessions after infusion of the deficient factor; early passive mobilization without adduction and limiting intra- and extra-rotation; isometrics exercises; progressive recovery of weight bearing and gait exercises with canes or crutches.

## 5. Total Hip Arthroplasty: Results and Complications

THA is a successful procedure for haemophilic patients with dramatic improvements regarding pain relief and function recovery. On the other hand, persons with haemophilia are not easy patients and it is a demanding procedure. Few large series have been reported with interesting clinical data.

The first long-term follow-up report was published by Luck et al.: 13 patients treated by cemented components They reported some failures mainly related to infections and aseptic loosening [24].

Kelley et al reported on 27 haemophiliacs (34 THA): 28 cemented and six cementless implants. At an 8-year follow-up for the cemented ones, the survival was 65% for acetabular components and 44% for femoral stems. For the cementless implants, there were only three years of postoperative evaluation [27].

Nelson et al. reported a series of 39 patients undergoing THA with a survival rate of 70% at an eight-year follow-up. The rate of complications was high: one patient died during surgery (cardiac arrest); one suffered a postoperative dislocation (managed by closed reduction); and five cases were revised early due to infections (two cases), and aseptic loosenings (three cases). Three additional cases showed a component migration with a scheduled revision [29]. 

Miles et al. reported a series of 30 persons with haemophilia treated by 26 cemented and four uncemented implants with MOP or COC couplings at more than six years of follow-up. They recorded a poor survival rate due to three deaths, three aseptic loosenings, and one deep infection [31]. 

**Figure 3:**
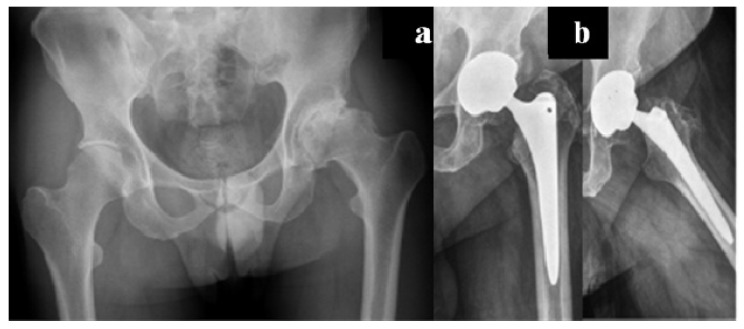
(**a)** Preoperative X-rays of the left hip of a 58-year-old patient affected by severe haemophilia A; (**b**) Radiographic aspect 6.5 years after surgery: Standard cementless tapered stem with ceramic-on-polyethylene (COP) coupling.

**Figure 4:**
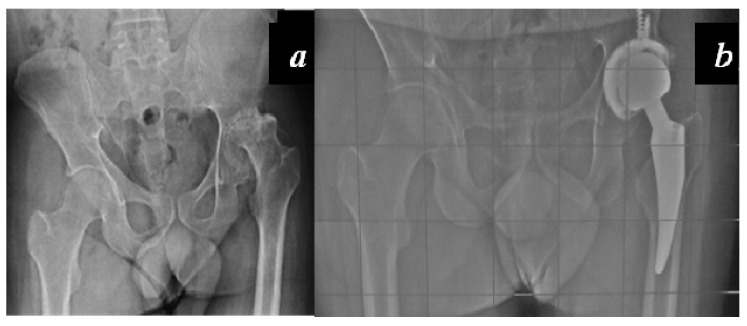
(**a**) Preoperative x-rays of the left hip of a 33-year-old patient affected by severe haemophilia A; (**b**) X-rays 3 years after surgery: cementless short stem, acetabular cup fixed with two supplementary screws and COP coupling.

Carulli et al. reported a series of 23 haemophilic patients undergoing THA with modern cementless implants and COP couplings [1]. In 14 subjects, a standard stem was used, while in nine cases a modern short stem was adopted. No failures and no complications were recorded, with a survival rate of 100% at an 8.1-year follow-up (Figure 3 and Figure 4).

Lee et al. reported a series of 21 cementless THA in 17 haemophilic subjects with a mean follow-up of more than ten years. The survival rate was 95.2% at that time. Three revisions were performed. [33].

**Figure 5:**
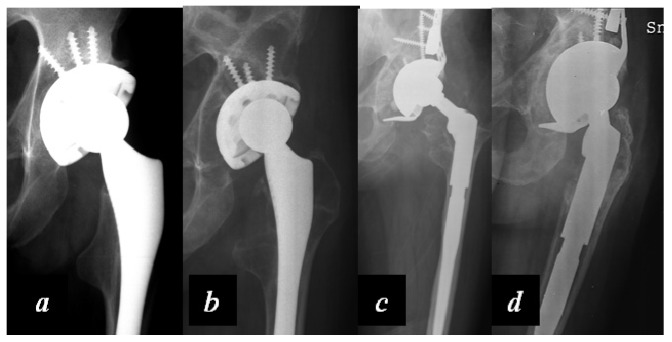
(**a**) Primary total hip arthroplasty (THA) of a 47-year-old severe haemophilia A patient; (**b**) Aseptic failure of the implant with loosening; (**c**) Revision with modular acetabular and femoral prostheses and acetabular reconstruction by biological composite (heterologous bone chips enriched with a concentration of autologous mesenchymal cells harvested from the iliac crest) and a trabecular metal wedge fixed by screws; (**d**) Four years after revision surgery, optimal integration of the components.

In the case of failure of a primary implant, the use of modular revision implants and the principles of tissue engineering may be very useful to obtain a good osteointegration and an adequate reconstruction of the joint (Figure 5).

## 6. Conclusions

Hip arthropathy in haemophilic patients is less common than in other joints but similarly disabling. Primary prophylaxis in subjects with moderate to severe factor deficiecy should be sufficient to prevent bleedings in the hip. However, persons with mild or moderate haemophilia may develop arthropathy. A preventive approach based on adequate prophylaxis, gentle physical activity, physical therapy after bleedings, and anti-inflammatory drugs may be proposed in the early stages of the disease, associated with strictly periodic musculoskeletal monitoring. 

In the case of persistent symptoms, injections with specific purposes may be proposed. Moderate to severe stages of hip arthropathy have to be addressed by a hip replacement. 

The use of modern and highly performant cementless implants, associated with a multidisciplinary approach to haemophilia, is the key to achieving good clinical outcomes, a low rate of complications, and a long-lasting prosthesis.
